# Discovery of Actinomycetes from Extreme Environments with Potential to Produce Novel Antibiotics

**DOI:** 10.5195/cajgh.2018.337

**Published:** 2018-12-14

**Authors:** Lyudmila Trenozhnikova, Azliyati Azizan

**Affiliations:** 1 Institute of Microbiology and Virology, Almaty, Kazakhstan; 2 Nazarbayev University School of Medicine, Astana, Kazakhstan

**Keywords:** Extremophiles, Actinomycetes, Antibiotics, Natural Products, Pathogens, Antibiotic Resistance

## Abstract

**Introduction:**

Antimicrobial-resistant pathogens pose serious challenges to healthcare institutions and health of the public. Thus, there is an urgent need for the discovery of new and effective antimicrobial agents. Microorganisms that exist in extreme environments such as those with high salinity or alkalinity, are known as extremophiles, and include various species of actinomycetes. The goal of this study is to discover novel antibiotics from extremophiles found in Kazakhstan that are effective against drug resistant pathogens.

**Methods:**

Soil from extreme environments of Kazakhstan was collected, and pure cultures of actinomycetes were isolated and cultured in modified Bennett’s broth with either high concentrations of salt or high pH to mimic extreme environments. Extracts obtained from selected actinomycetes strains were used to test for antimicrobial activity against *Staphylococcus aureus*, *Escherichia coli* and *Aspergillus niger*.

**Results:**

A total of 5936 strains of extremophile actinomycetes were isolated; from these, 2019 strains were further isolated into pure cultures. Of these, 415 actinomycetes strains that demonstrated antagonistic antibacterial activities were selected. These actinomycetes were further classified into groups and subgroups based on their responses to different culture conditions. Antimicrobial antagonism activity for some of the actinomycetes strains was dependent on culture conditions and development of aerial mycelia under extreme conditions.

**Conclusion:**

We identified several interesting candidate extracts with putative antimicrobial activities against several strains of drug resistant pathogens. Our research of the actinomycetes’ ability to produce antibiotics in the near-natural conditions provides a great opportunity to assess their biodiversity and distribution in the Central Asian region and to develop new methodological approaches to the screening of new antimicrobial agents.

## Introduction

Although antibiotics have saved millions of lives over the past 70 years, their indiscriminate use has led to the emergence of antibiotic-resistant organisms. This concerns many medical experts who caution that we may soon return to the pre-antibiotic era^1^,^2^.

Actinomycetes, which occur in both terrestrial and aquatic habitats, are among the most common groups of gram-positive microorganisms in nature. Actinomycetes decompose organic matter and display antagonism against other bacteria and fungi, with which they compete for nutrients. Actinomycetes have incredible abilities to survive under extreme conditions in their natural environment and have long been the focus of scholarly attention and have been harnessed as valuable sources of natural compounds, such as antibiotics, enzymes, and vitamins. More than 90 percent of chemotherapeutic antibiotics have been isolated from actinomycetes^3^–^5^.

The large-scale screening of antagonistic actinomycetes under neutral conditions (with a pH of 7.0 and no addition of salt [NaCl, Na_2_CO_3_] to the growth media) has led to the discovery of virtually all modern medical antibiotics^1^,^2^. During screening, the strains of actinomycetes that did not show activity in neutral environments were likely disregarded. Microorganisms that produce antimicrobials under extreme growth conditions may have gone undetected while being screened under neutral growth conditions, preventing the discovery of potentially valuable novel compounds from these strains.

Advances in PCR technology have revealed that actinomycetes do not always exhibit antagonistic properties when cultivated *in vitro* despite the presence of genes that are involved in antibiotic synthesis in the genome of the strain. This suggested that dormant genes may be present but not expressed. Certain natural conditions may exist under which these genes are expressed, and identification of these conditions is important when screening for the new natural antibiotic substances. Modern screening programs should take into account the relevant characteristics of the actinomycetes and should also include alternative conditions for antibiotic isolation. To that end, we undertook a study utilizing an alternative method of screening to enhance the chances of discovering unique bacterial strains from the environment that are potentially producing novel antibiotics for therapeutic use.

This study focuses on actinomycetes strains isolated from the extreme habitats of Kazakhstan. We investigated the characteristics of actinomycetes in the three most commonly occurring natural habitats/conditions (which are neutral, saline, and alkaline). We believe that this approach, which is different than the conventional screening methodology, enhances the chance of identifying actinomycetes strains from extreme environments that harbor the potential to produce novel antimicrobial agents. Kazakhstan possesses unique natural resources that can be sources of promising and novel biologically-active substances with antibiotic properties. Soil and water resources of the Republic of Kazakhstan provide a great variety of natural habitats for extremophilic microorganisms (such as solonchaks, solonetzes, and saline takyrs). These habitats differ both in morphological features and salinization (chloride, sulfate, soda, and mixed). The water resources of Kazakhstan present a wide variety of habitats for extremophiles; this includes the Caspian Sea, the Aral Sea, Balkhash Lake, and numerous saline lakes in which water salinity can reach up to 335 g/l. The area of saline soils in Kazakhstan (including solonetzes, alkaline soils and combinations with other soils) is 111.55 million hectares (0.4307 million square miles), or 41% of the entire territory of the country (1.052 million square miles)^6^. Most of these areas are marked by natural mineralization due to the presence of marine sediments. Overall, research on extremophilic microorganisms in Kazakhstan has been limited. The goal of this publication is to describe the process of screening for antagonistic activity against drug-resistant pathogens and characterize actinomycetes strains which are the potential producers of novel antibiotics.

## Materials and methods

### Sampling sites and collection of soils

Natural substrate samples (soils, muds, and rhizosphere) were collected from the extreme habitats of Northern (Kostanay region, Auliekol and Mendykara district) and Southern (Almaty region, Balkhash district) Kazakhstan during several field trips in summer 2010–2011 (Fig. 1A). The soil samples collected were of the solonchaks, solonetz and takyr types. Solonchaks are strongly saline soils, which are usually light colored and are typically developed in poorly drained arid or semiarid areas vegetated mostly by halophytes^7^. Solonetz are soils with a high content of exchangeable sodium and/or magnesium ions^8^. Takyr is usually formed in a shallow, depressed area with a heavy clay soil which is submerged by water after seasonal rains; after the water evaporates, a dried crust with fissures forms on the surface^9^. The soil samples were collected at the depth of 10 cm (~4 inches). The samples were packed in sterile plastic containers, transported to the laboratory, and refrigerated at 4°C (39°F) until ready for analysis. Natural substrate samples (soils, muds, and rhizosphere) were collected from the extreme habitats of Northern and Southern Kazakhstan during several field trips in the summer of 2010–2011.

We chose the most common factors in nature, which are sodium chloride and pH, and grew the same actinomycetes in different growth media to mimic the natural environment of extremophiles. The conditions these organisms were originally isolated from are neutral, saline, and alkaline environments. Strains of actinomycetes from extreme environments of Kazakhstan were cultured in neutral and alternative (saline and alkaline) conditions and their antimicrobial and morphogenetic properties were studied. Saline and alkaline conditions were created by using inorganic salts (NaCl, Na_2_CO_3_) and pH modulation.

### Isolation and maintenance of Actinomycetes

Soil samples were plated following the standard microbiological dilution plating method. Actinomycetes samples were isolated on two variants of modified Bennett’s agar: glucose (0.2%), peptone (0.2%), yeast extract (0.1%), and agar (2.0%) with 5% NaCl, pH 7,2 (variant #1) or with 0.5% Na_2_CO_3_, pH 9.0 (variant #2). The medium was adjusted with NaOH to pH 9.0 after sterilization.

The plates prepared with variants of modified Bennett’s agar were incubated at 28°C and examined for growth after 1–2 weeks of incubation. The colonies with different cultural-morphological characteristics were inoculated from the variants of modified Bennett’s agar (variant #1 and variant #2) into slants with the same variants of the medium. The purity of isolated strains was confirmed by standard microbiological methodologies whereby strains were isolated in pure cultures from separately growing colonies obtained from plates seeded with soil samples. Each colony was further tested by screening on specific agar growth media as described.

Purified isolates were maintained on the variants of modified Bennett’s agar slants at 4°C for further antagonism tests and morphogenesis investigation. The percent of the total number of strains isolated in the relevant region was determined.

### In vitro antimicrobial assay

Actinomycetes isolates were tested for their antagonistic activity against the selected microorganisms by the disc diffusion agar method^10^.

#### The first stage of screening**:**

The isolates were cultured on the variants of modified Bennett’s agar (#1 or # 2) for 10 days at 28°C. Agar discs (7 mm) were cut by a cork borer and transferred to the surface of agar plates, previously inoculated with bacterial test organisms (hospital strain *MRSA* # 3316). The petri dishes were kept in a refrigerator for 3 hours before incubation to permit the diffusion of antimicrobial substances. The diameters of the inhibition zones were measured after incubation for 24 hours at 37°C.

#### The second stage of screening

The isolates with antagonistic properties, selected based on the results of the first screening stage, were cultured on three variants of modified Bennett’s agar (#1–3) for 10 days at 28°C. Variant # 3 of modified Bennett’s agar corresponded to a neutral habitat: glucose (0.2%), peptone (0.2%), yeast extract (0.1%), and agar (2.0%) at pH 7.2. Agar discs (7 mm) were cut off by a cork borer and transferred to the surface of agar plates, previously inoculated with the test organism (*MRSA* # 3316, *Escherichia coli* pMG223, and *Aspergillus niger*). The petri dishes were kept in a refrigerator for 3 hours before incubation to permit the diffusion of antimicrobial substances. The diameters of inhibition zones were measured after incubation for 24 hours at 37°C for bacteria and for 72 hours at 28°C for fungi. Each test was repeated three times and the activity was expressed as the mean diameter of the inhibition zones (mm).

### Study of morphogenesis

The degree of aerial mycelium development in isolates of actinomycetes was studied visually on three variants of modified Bennett’s agar (#1–3).

### Bacterial and fungal pathogens

The following bacterial strains were used in this study as testers for the antimicrobial activity of the isolated actinomycetes strains: hospital strain *MRSA* # 3316, *Escherichia coli* (pMG223) and *Aspergillus niger* (wildtype isolate).

## Results

In Northern Kazakhstan (Kustanai region), samples were collected from the soils of the steppe and forest zones, sor solonchaks (non-perennial salt lakes in the area of Aman-Karagai forest), solods, solonets (steppe and meadow), rhizospheres of plants from halophytic meadows, and muds from the salt lakes (Fig. 1B). In Southern Kazakhstan (Almaty region), samples were collected from the soils of the arid zone, typical meadows and sor solonchaks, salinized takyrs, takyr-like salinized soils, and rhizospheres of the arid zone plants (Fig 1C). We collected 36 samples of natural substrates from extreme ecosystems in Northern Kazakhstan and 50 samples from extreme ecosystems in Southern Kazakhstan.

We identified a link between the ability of actinomycetes to grow under conditions reflective of three habitats, the ability to antagonize in each of these conditions, and the ability to form aerial mycelia. In the course of our research, we focused on three main types of ecological niches: neutral habitats (pH 7.0), saline habitats (pH 7.0), and alkaline habitats (pH 9.0).

A total number of 5936 actinomycetes strains were isolated on the two variants of modified Bennett’s agar; from these, 2019 strains of extremophile actinomycetes grew in media #1 and #2 and differed in cultural-morphological characteristics. These strains were further isolated in pure cultures (756 strains from Northern Kazakhstan and 1263 strains from Southern Kazakhstan). The actinomycetes from the extreme environments of Kazakhstan were analyzed based on their ability to show antagonism against *MRSA* in saline or alkaline conditions. A total 415 strains with antagonistic properties were selected: 127 strains from Northern Kazakhstan and 288 strains from Southern Kazakhstan. These strains showed antagonistic properties against bacterial pathogens when grown under extreme conditions in media # 1 and #2. 100 percent of these 415 strains showed antagonism against clinical *MRSA* # 3316, 21.6 percent against *E. coli* (pMG223), and 28.4 percent against *A. niger*.

The correlation of the changes in growth, morphogenesis, and antagonism of 415 strains of extremophile actinomycetes was determined under three conditions, modeling the most common natural habitats: neutral, saline, and alkaline. The actinomycetes were further classified into groups, subgroups, and variants (Table 1) based on their ability to antagonize (exhibiting antimicrobial activities of test organisms MRSA, *E. coli* and *A. niger*) in all three habitats (Subgroup IA), only two habitats (Subgroup IB), or only one habitat (Subgroup IC).

Subgroups IIa showed growth in neutral and saline conditions, IIb showed growth in neutral and alkaline conditions, and IIc showed growth in saline and alkaline conditions.

**Figure 2:**
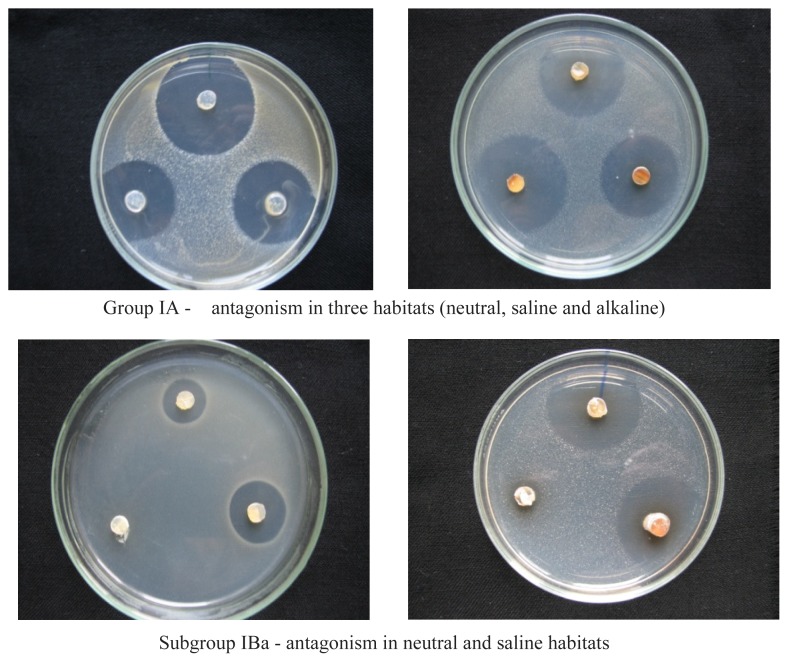

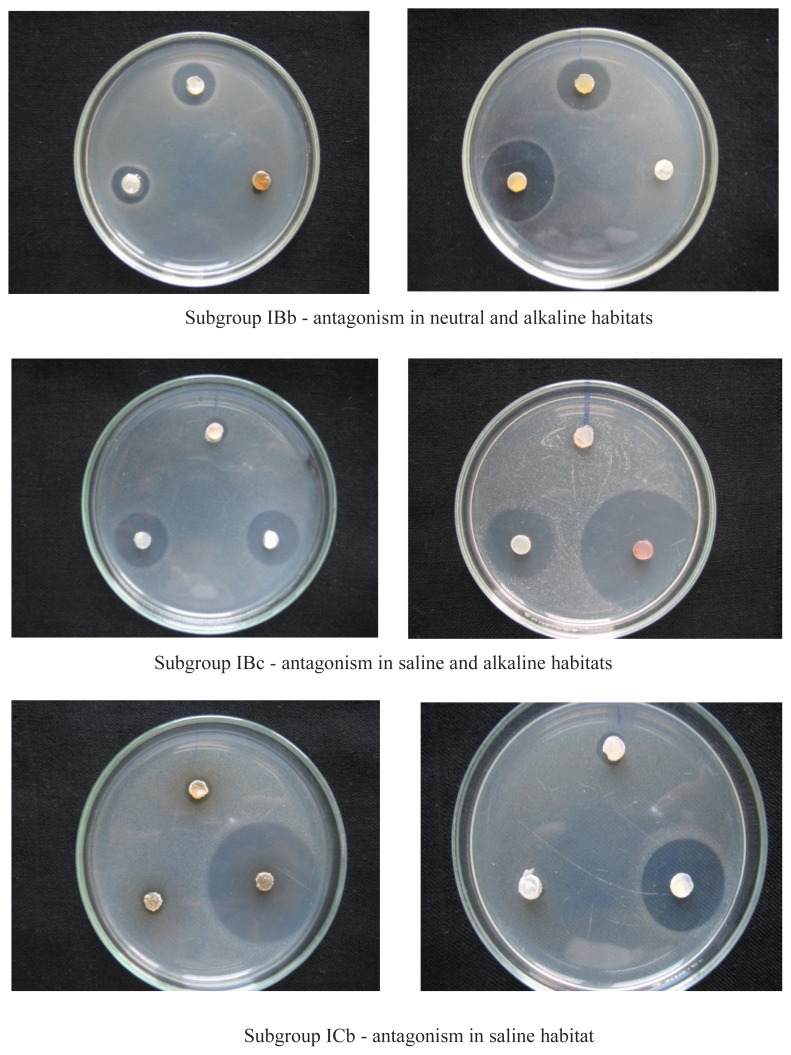

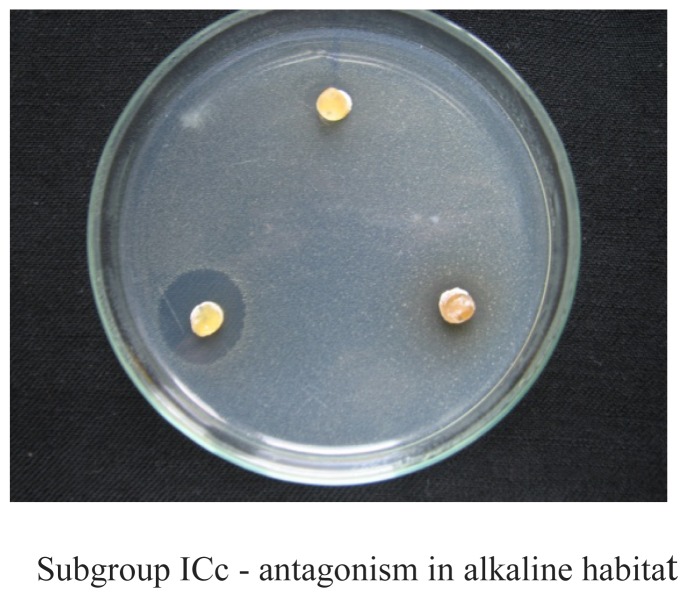
The classification of actinomycetes from extreme ecosystems into groups and subgroups

In each group, we established the following subgroups. Three subgroups were identified in Group I: IA, IB, and IC (Fig. 2). The subgroups of I are as follows; IA subgroup – actinomycetes that show antagonism in three conditions (neutral, saline and alkaline): IB subgroup – actinomycetes that show antagonism in two conditions: IBa – antagonism in neutral and saline conditions: IBb – antagonism in neutral and alkaline conditions: IBc – antagonism in saline and alkaline conditions: IC subgroup – actinomycetes that show antagonism in one medium: ICa - antagonism in neutral conditions: ICb – antagonism in saline conditions, and ICc – antagonism in alkaline conditions.

Two subgroups were identified in Group II: IIA and IIB. The subgroups of Group II are as follows; IIA subgroup – actinomycetes that show antagonism in two media: IIAa – antagonism in neutral and saline conditions: IIAb – antagonism in neutral and alkaline conditions, and IIAc – antagonism in saline and alkaline conditions: IIB subgroup – actinomycetes that show antagonism in one medium: IIBa – antagonism in neutral conditions, IIBB – antagonism in saline conditions, IIBc – antagonism in alkaline conditions.

In summary, the groups of actinomycetes differ from one another in their ability to grow in different habitats, which are neutral, saline and alkaline. We define subgroups by the ability to produce antibiotics, or they may lose this ability to produce antibiotics in these specific habitats (neutral, saline or alkaline). The magnitude of the inhibition zone was determined by us, but for this study, only the presence or total absence of antibiotic production was important for the classification of actinomycetes that we identified in this study. Since it was important to determine the conditions under which antibiotic production is possible for each of the producer organisms, the strains were classified into these specific groups, subgroups and variants.

Table 2 summarizes the data on the quantitative content of actinomycetes-antagonists of different group I subgroups in conditions mirroring those of Southern and Northern Kazakhstan. The data on the actinomycetes of Subgroup II is not discussed here, as the group did not have a sufficient size to be represented accurately.

We observed differences in the occurrence of Group I antagonists in the natural substrates of the Southern and Northern Kazakhstan. The antagonism against gram-positive bacteria in both researched regions is characterized by the predominance of Subgroup IA (55.4 – 72.3%); Subgroup IB was less common (21.0 – 34.0%), while Subgroup IC had the smallest amount of antibacterial activity against gram-positive bacteria (6.7 – 10.6%). The antagonism against gram-negative bacteria and mycelial fungi also varied by region. In Southern Kazakhstan, the Subgroup IA prevailed (41.2 – 41.4%), while the Subgroups IB and IC occurred less commonly. In Northern Kazakhstan, the antagonism against gram-negative bacteria was characterized by the predominance of the Subgroups IA (41.7%) and IC (38.9%). The antagonism against mycelial fungi in the researched substrates of Northern Kazakhstan is characterized by the predominance of the Subgroup IB (40.7%), while in Southern Kazakhstan, Subgroup IA predominates (41.4%).

Our studies of the dependence of antagonism on the degree of development of the aerial mycelium of actinomycetes under extreme conditions show that actinomycetes can be represented by the two main variants, which we designated as F and Q (Table 3) whereby “F” represents the “Fighters” and “Q” represents the “Quitters” which we describe further below.

The variant Q is represented by the actinomycetes whose antagonism is associated with good growth and abundant development of the aerial mycelium. Reduction of sporulation leads to a decrease in the production of antibiotics, and absence of aerial mycelium to the loss of this ability.

The variant F is represented by the actinomycetes that exhibit antagonistic properties only in the conditions when the formation of aerial mycelium is inhibited, and its absence is associated with the maximum formation of antibiotics.

The actinomycetes from the extreme environments of Kazakhstan were analyzed based on their ability to show antagonism against *MRSA* in saline or alkaline conditions. The isolates with antagonistic properties, selected based on the results of the first screening stage, were cultured on three variants of modified Bennett’s agar (#1–3) for 10 days at 28°C. Variant #1 mimics a saline habitat, while variant # 2 mimics an alkaline habitat and variant # 3 mimic neutral habitats. Agar discs (7 mm) were cut by a cork borer and transferred to the surface of agar plates, previously inoculated with the test organism (*MRSA* # 3316, *Escherichia coli* pMG223, and *Aspergillus niger*). The diameter of inhibition zones was measured after incubation for 24 hours at 37°C. Each test was repeated three times and the activity was expressed as the mean of diameter of the inhibition zones (mm). The actinomycetes were classified into these groups; I (growth in all three habitats) and II (growth in two habitats). The actinomycetes group I, showing the ability to grow in all researched habitats, had a greater variability of antagonistic properties. These actinomycetes groups were further classified into subgroups based on their ability to antagonize in the saline, alkaline and neutral habitats. Group IA - antagonism in three habitats (neutral, saline and alkaline). Subgroup IBa - antagonism in neutral and saline habitats. Subgroup IBb - antagonism in neutral and alkaline habitats. Subgroup IBc *-* antagonism in saline and alkaline habitats. Subgroup ICb - antagonism in saline habitat. Subgroup ICc - antagonism in alkaline habitat.

## Discussion

We have identified 415 strains of actinomycetes that demonstrate varying degrees of antifungal and antibacterial activities in saline and alkaline habitats. Some of these strains may be producing previously unknown antibiotics.

Traditionally, researchers have studied the diversity of actinomycetes using neutral growth media. Similarly, isolation, research of their properties, and production of natural, biologically active substances have been associated with strains grown in neutral media and conditions. These neutral conditions served as the basis of the discovery of contemporary natural antibiotics. However, the prevalence of drug resistance to known antibiotic substances necessitates the expansion of the boundaries of screening and changes in screening methods in order to identify new and promising antimicrobials^11^,^12^. Thus, unusual natural substrates and extreme ecosystems have currently become the most popular targets of research as they are the most likely to yield new microorganisms with unique properties. Marine environments, as well as soils with high levels of salinity and alkalinity, are being actively studied as sources of new secondary metabolites^13^ With their unique metabolic pathway formed in special environments, microorganisms from extreme environments produce many special bioactive substances, such as enzymes and antibiotics^14^–^17^. The molecules formed by extremophilic microorganisms have unique properties that offer ample opportunities for a variety of applications^18^–^22^.

There are several limitations in this study. When investigating antagonism under changing conditions, we did not conduct studies in an acidic environment. In addition, in nature there may be other growth conditions that are not represented by the three media that we used to grow actinomycetes in the laboratory, therefore we may have missed the detection of other potential antibiotics produced by actinomycetes. The screening does not take into account the possibility of re-discovery of known antibiotics from certain strains, therefore we plan to develop approaches to rule out this possibility in future studies. The strains studied were not identified at the level of genus and species; however, in future studies we plan to perform these studies using traditional microbiology methods as well as molecular (PCR) methodologies.

It is important to determine how actinomycetes interact with their changing environment and with other microorganisms in relation to their ability to consume nutrients and produce antibiotics. The main characteristics of interest of actinomycetes from extreme environments of Kazakhstan are their growth, ability to form aerial mycelia, and their ability to antagonize bacteria and fungi.

We have determined that the most widespread antibiotic-producing organisms were the actinomycetes belonging to group I; they grew in each of the selected habitats – neutral, saline, and alkaline. This important characteristic accounts for the wide distribution of the actinomycetes in soils and water. These actinomycetes also constituted the majority of those isolated in our research. Actinomycetes of group II were very rare. When tested for growth in three different conditions (neutral, saline or alkaline), all strains grew in at the least two or three conditions. We did not find any isolate that grew only in one condition. Their absence may be a result of the isolation method used. However, their absence also indicates that despite their isolation from extreme habitats, actinomycetes are highly adaptable to different environmental conditions, including the neutral media. Thus, screening from any extreme natural substrates, including the less explored marine sources, will enable the isolation of actinomycetes that belong to the subgroups IA, IBa, IBb, which grow and secrete antimicrobials in neutral conditions and, perhaps also, secrete already known antibiotic compounds. Our proposed classification allows researchers to concentrate their efforts on studying antibiotic-producing actinomycetes that belong to the specific groups IBc, ICb, ICc, IIAc, IIBb, and IIBc, which do not show antagonism under neutral conditions but do show antagonism under saline and/or alkaline conditions; these actinomycetes have the greatest potential for yielding new and unexplored antibiotic compounds.

Actinomycetes are an unusual group of gram-positive bacteria that have differentiated mycelia, both substrate and aerial. Information on the relationship of morphogenesis and antibiotic formation in actinomycetes is often contradictory. Some studies have correlated initial stages of antibiotic production with inhibition of aerial mycelium formation, while others indicate that the formation of antibiotics is linked to its abundant formation^23^–^28^. We found two variants of actinomycetes whose antagonism is inversely related to their morphogenesis. In the actinomycetes of the variant Q “quitters” (which constituted 39.8% of total actinomycetes isolated), antagonism was correlated to good growth and formation of aerial mycelium. The reduction of sporulation resulted in a decrease of antibiotic production, and its absence resulted in the loss of this ability. Actinomycetes of the variant F “fighters” (60.2% of total actinomycetes) exhibited antagonistic properties only when growth was poor and the production of aerial mycelium was inhibited, and its absence was associated with the highest level of antagonism. Accordingly, in each previously selected subgroup based on the type of antagonism exhibition, the actinomycetes may be represented by these variants. Actinomycetes of the variant Q are more attached to certain habitats that ensure their comfortable existence, while the actinomycetes of variant F are capable of freer movement in a variety of habitats and colonization of new environments. We can conclude that in any natural environment members of both groups can be present simultaneously and can engage in certain relationships that ensure the survival of the actinomycetes community as a whole.

The research of the actinomycetes’ ability to produce antibiotics in near-natural conditions provides a great opportunity to assess their biodiversity and distribution in the various regions of the world and to develop new methodological approaches to the screening of new antibiotics. Those groups of actinomycetes that produce antibiotics only under extreme conditions, even if these are neutrophile strains of the variant F, may be the most promising for modern screening. Once their growth is inhibited, the neutrophiles of the variant F may produce previously unknown antibiotics in saline and alkaline habitats. We consider the following subgroups to hold the greatest potential for screening: IBc, ICb, ICc, IIAc, IIBb, and IIBc. In future studies, we hope to further characterize the nature of these unknown antibiotics and to test them against known fungal and bacterial pathogens, particularly those strains that may be resistant to antibiotics currently available in the market. Additionally, one of our long-term goals of this work is to identify known strains of actinomycetes that exhibit interesting characteristics to the species level (which would be relevant to this study particularly for strains that produce potentially novel antibiotics), and/or to potentially identify novel actinomycetes strains.

An organism’s environment may vary greatly, and a neutral habitat may gradually become saline, alkaline or acidic. Accordingly, actinomycetes ability to survive is largely determined by their adaptability. The study of the behavior of actinomycetes, which use antibiotics for their survival in changing habitats may allow for the development of new approaches to the identification of pharmaceutically valuable drugs. This study has a significant global health implication. The search for new antibiotic-producing strains will require collaborations of many countries and will significantly enhance global health if new antibiotic agents become available. The actinomycetes classification we propose here is neither complete nor indisputable, and it can be changed and supplemented with new data and hypotheses that make it possible to significantly expand it.

## Figures and Tables

**Figure 1A:**
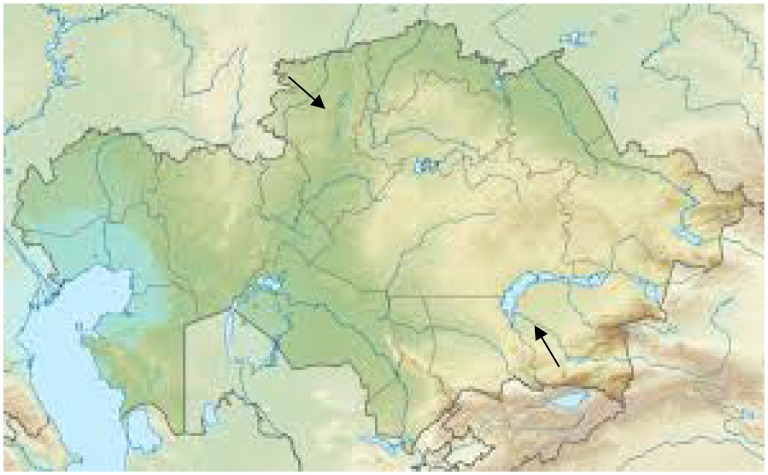
Map of field trips collecting soil samples

**Figure 1B:**
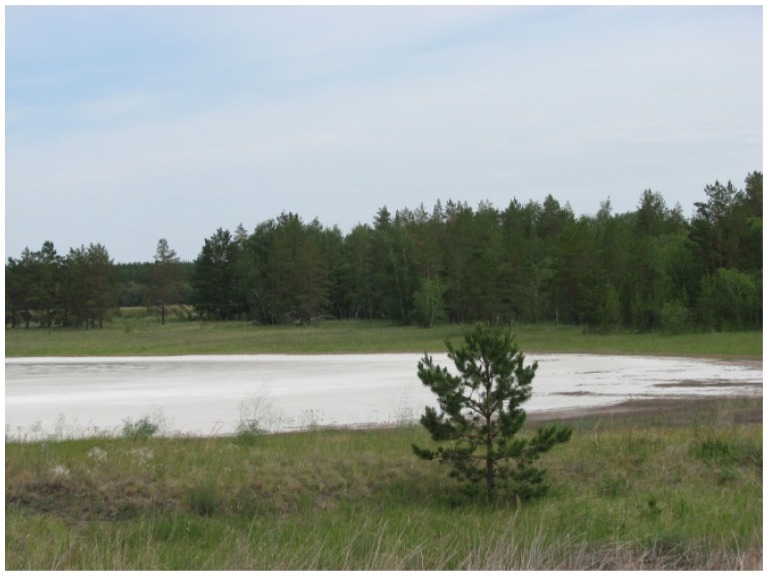
The unusual ecosystem of Northern Kazakhstan. In Northern Kazakhstan (Kustanai region), samples were collected from the soils of the steppe and forest zones, sor solonchaks (non-perennial salts lakes in the area of Aman-Karagai forest), solods, solonets soils (steppe and meadow), rhizospheres of plants from halophytic meadows, and muds from the salt lakes.

**Figure 1C:**
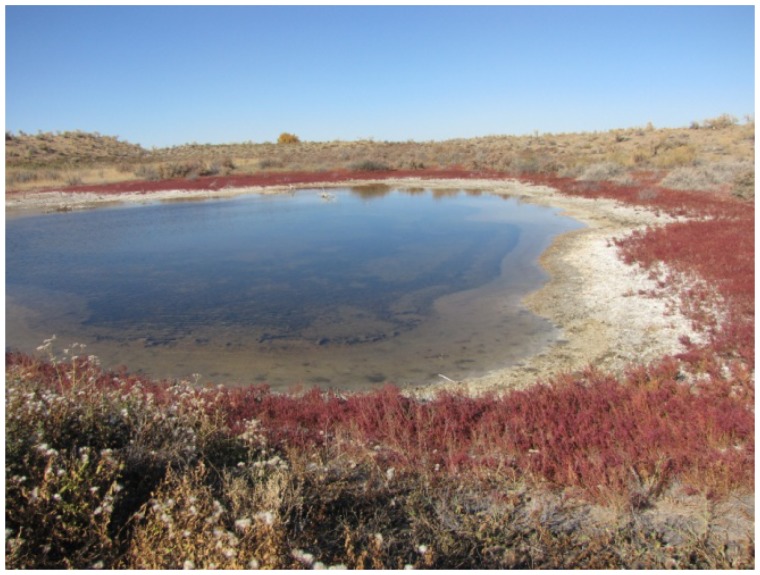
The unusual ecosystem of Southern Kazakhstan. In Southern Kazakhstan (Almaty region), samples were collected from the soils of the arid zone, typical meadows and sor solonchaks, salinized takyrs, takyr-like salinized soils, and rhizospheres of the arid zone plants.

**Table 1:** Classification of actinomycetes based on their ability to grow under different conditions

Group of actinomycetes	Growth in neutral habitat	Growth in saline habitat	Growth in alkaline habitat
I (all habitats)	+	+	+
II (two habitats)			
IIa	+	+	−
IIb	+	−	+
IIc	−	+	+
+ means growth; − means no growth			

**Table 2:** The quantitative content of Group I antagonists in the extreme ecosystems of Kazakhstan

Subgroups of actinomycetes	Quantitative content of antagonists, %^1^		
	Antibacterial activity (gram-positive bacteria, *S. aureus*)	Antibacterial activity (gram-negative bacteria, *E. coli*)	Antifungal activity (*A. niger*)
2Southern Kazakhstan	N = a	N = b	N = c
Subgroup IA	55.4	41.2	41.4
Subgroup IB	34	37.3	29.3
Subgroup IC	10.6	21.5	29.3
3Northern Kazakhstan	N′ = x	N′ = y	N′ = z
Subgroup IA	72.3	41.7	29.6
Subgroup IB	21	19.4	40.7
Subgroup IC	6.7	38.9	25.9

1 Note – Total activity for the three subgroups IA, IB, and IC add up to 100%, for each kind of antibacterial or antifungal activity tested.

2 Note-Total number (N) of actinomycetes strains from Southern Kazakhstan tested was 1,263.

3 Note-Total number (N) of actinomycetes strains from Northern Kazakhstan tested was 756.

**Table 3:** The quantitative content of variants of actinomycetes in the extreme ecosystems of Kazakhstan

Type of soil	Variants of actinomycetes	
	Variant F^1^	Variant Q^2^
Solonchaks	67.2	32.8
Takyr-like salinized soils	56.7	43.3
Salinized takyrs	51.8	48.2
Solonets soils	50.4	49.6
Solods	75.0	25.0
Total	60.2	39.8

1 F (“Fighters”) – antagonism in the absence of aerial growth (i.e., when they are fighting to survive);

2 Q (“Quitters”) – antagonism associated with good aerial growth (i.e., quit producing antibiotics when growth is poor)
